# Our Original Department

**Published:** 1874-05

**Authors:** 


*Our Original Department*, it will be seen, covers the larger portion of this number. We think our readers will be pleased with the character as well as the number of articles published. We desire to say to all our friends that we hope they will not cease to write because they may suppose we are crowded with articles. That we are crowded is true, but we *will always make room for them*—even if we have occassionally, as in this issue, to give up the larger portion of our pages for the purpose. We therefore repeat our invitation to all—*write.* G.

